# Two Case Reports of Percutaneous Intravascular Lithotripsy for the Treatment of Severe Coronary Artery Calcification

**DOI:** 10.1002/ccr3.72834

**Published:** 2026-06-04

**Authors:** Liang‐cai Guo, Chi‐jun Hou, Yan‐jin Zeng, Wen Li

**Affiliations:** ^1^ Cardiovascular Department Dongguan Hospital of Traditional Chinese Medicine Dongguan Guangdong China

**Keywords:** angioplasty, coronary calcification, coronary stenosis, intravascular lithotripsy, percutaneous coronary intervention

## Abstract

Severe coronary calcification is a common challenge in percutaneous coronary intervention, often resulting in suboptimal stent expansion. Intravascular lithotripsy (IVL) employs acoustic pressure waves to fracture calcified plaques and increase procedural success. Two women, aged 85 and 76 years, with heavily calcified coronary lesions, underwent IVL prior to stent implantation. Post‐procedure intravascular ultrasound confirmed effective calcium modification and optimal stent expansion (< 10% residual stenosis). No perioperative complications occurred. Both patients remained symptom‐free at the 1‐year follow‐up.

## Key Clinical Message

Intravascular lithotripsy is an effective and safe strategy for modifying severe circumferential coronary calcification resistant to conventional balloon techniques, enabling optimal stent expansion and favorable short‐term outcomes in elderly patients undergoing PCI.

## 1 Introduction

Coronary calcification poses a significant challenge in percutaneous coronary intervention (PCI). Severe calcification exacerbates coronary stenosis, reduces vessel compliance, and impedes device delivery, thereby increasing procedural complexity. Additionally, calcification may damage stent polymer coatings, leading to suboptimal stent expansion and malapposition [1, 2]. Incomplete stent expansion is an important risk factor for in‐stent thrombosis and in‐stent restenosis, increasing the incidence of major adverse cardiovascular events (MACE) [3]. At present, special balloon dilatation, laser plaque ablation, orbital rotary resection, plaque rotary grinding, and other techniques have been gradually applied, but these techniques have their own limitations [4]. Intravascular lithotripsy (IVL) uses sound pressure waves generated at the calcified interface to selectively fracture severe calcific plaques—including both superficial and deep layers—thereby improving vascular wall compliance [5]. In principle, IVL can effectively treat calcified lesions by delivering pulse waves through the catheter–balloon system to selectively fracture hard calcified plaques beneath the intima without damaging the vascular wall and adjacent soft tissue [6], making it highly effective and safe. Complication rates during treatment are low, and clinical trials have demonstrated its effectiveness and safety in the pretreatment of severe calcified lesions [7], with favorable clinical outcomes reported during mid‐term follow‐up [8, 9]. Therefore, IVL has become an important approach for managing coronary artery calcification.

## 2 Case Presentations

### 2.1 Case 1

An 85‐year‐old woman was admitted to our hospital on April 30, 2024, with a 2‐week history of recurrent dizziness and chest tightness. Her medical history included uncontrolled hypertension.

#### 2.1.1 Diagnostic Evaluation

Vital signs included temperature 36.2°C, pulse 74 bpm, respiratory rate (RR) 20 breaths/min, blood pressure (BP) 144/73 mmHg. The patient was alert and oriented. Cardiovascular examination revealed regular rhythm and no murmurs. Her lungs were clear bilaterally. Her abdomen was soft, nontender, and with no organomegaly. Neurological and musculoskeletal examinations revealed no edema or deficits. Laboratory tests, including complete blood count, renal/liver function panels, electrolytes, coagulation profile, D‐dimer levels, cardiac troponin I levels, and pro‐BNP levels, were within normal limits.

#### 2.1.2 Cardiac Studies

**FIGURE 1:**
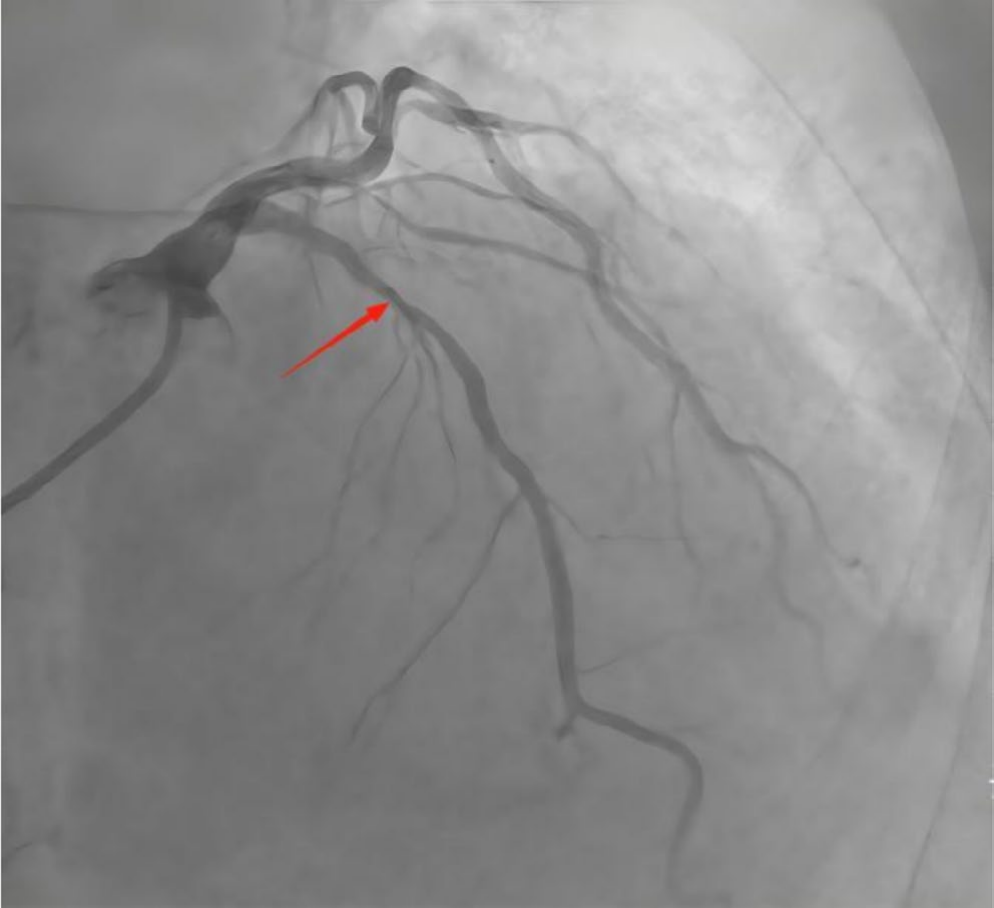
Coronary angiography image prior to intravascular lithotripsy, demonstrating severe calcification and vessel narrowing in the proximal left anterior descending artery.

**FIGURE 2:**
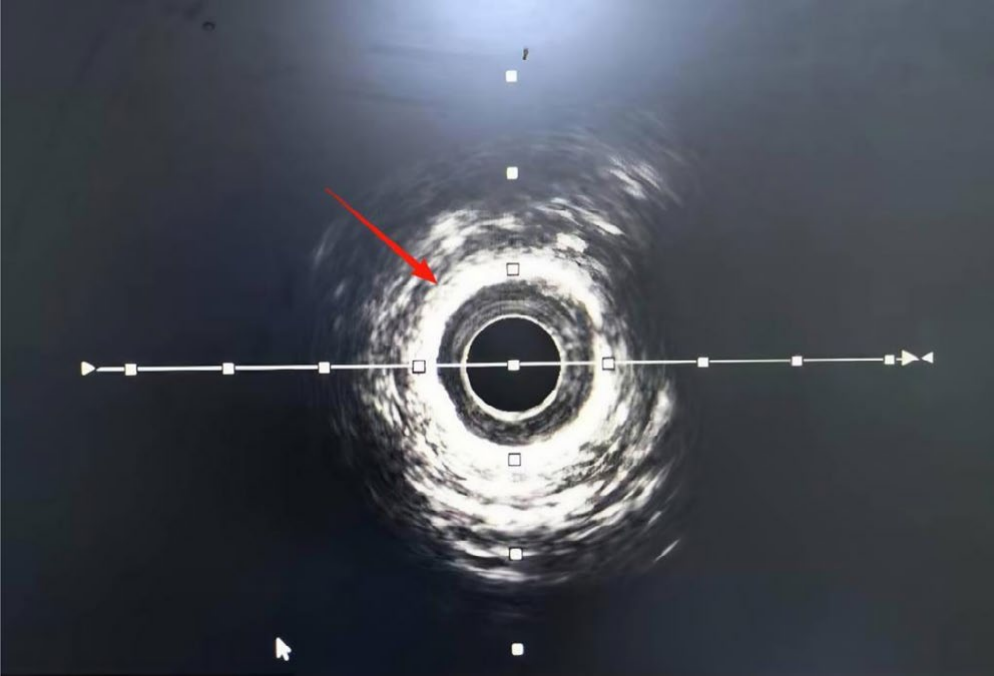
360° circumferential coronary calcification observed using intravascular ultrasound. The bright, continuous echogenic ring encircling the lumen reflects extensive calcific plaque, indicating a rigid vessel wall before treatment.

Electrocardiography (ECG) revealed a sinus rhythm and no ST‐T changes. Holter monitoring showed frequent premature ventricular contractions. Transthoracic echocardiography (TTE) revealed cardiac arrhythmia, mild mitral/aortic regurgitation, left ventricular diastolic dysfunction, and an ejection fraction of 54%. Coronary angiography revealed diffuse calcified 85% stenosis in proximal/mid segments of the left anterior descending (LAD) (Figure 1); left circumflex (LCX), 40%–50% proximal stenosis, 50% ostial obtuse marginal stenosis; right coronary artery (RCA), 50%–60% proximal stenosis, 60% mid‐stenosis. Intravascular ultrasound (IVUS) revealed 360° annular calcification in the LAD proximal/mid segments, with minimum lumen area 2.66 mm^2^ (71% plaque burden) (Figure 2).

#### 2.1.3 Intervention

**FIGURE 3:**
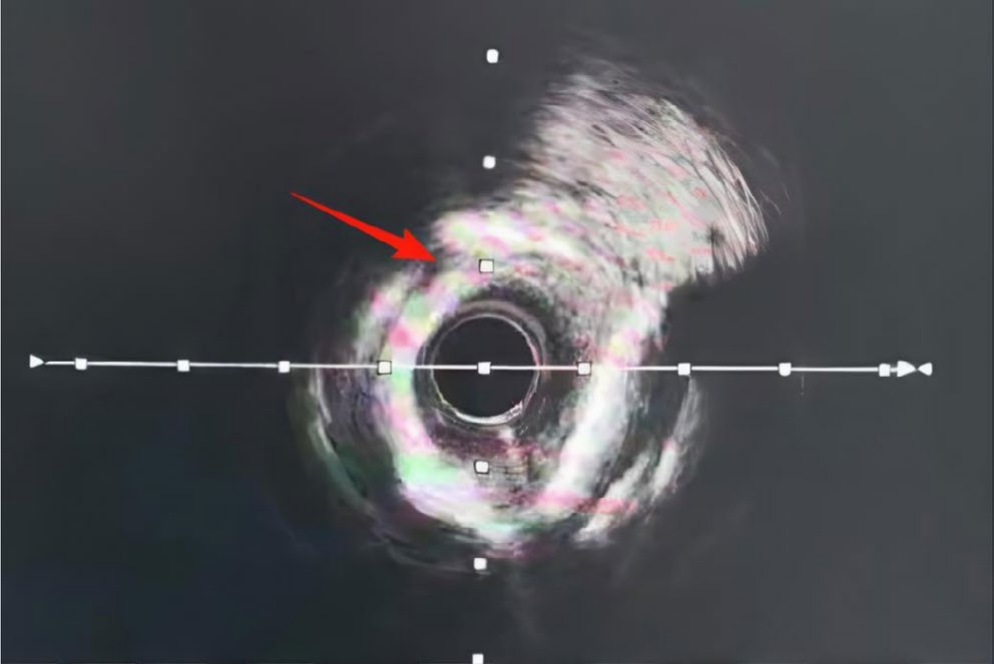
Destruction of calcified plaque after intravascular lithotripsy, as visualized on intravascular ultrasound.

**FIGURE 4:**
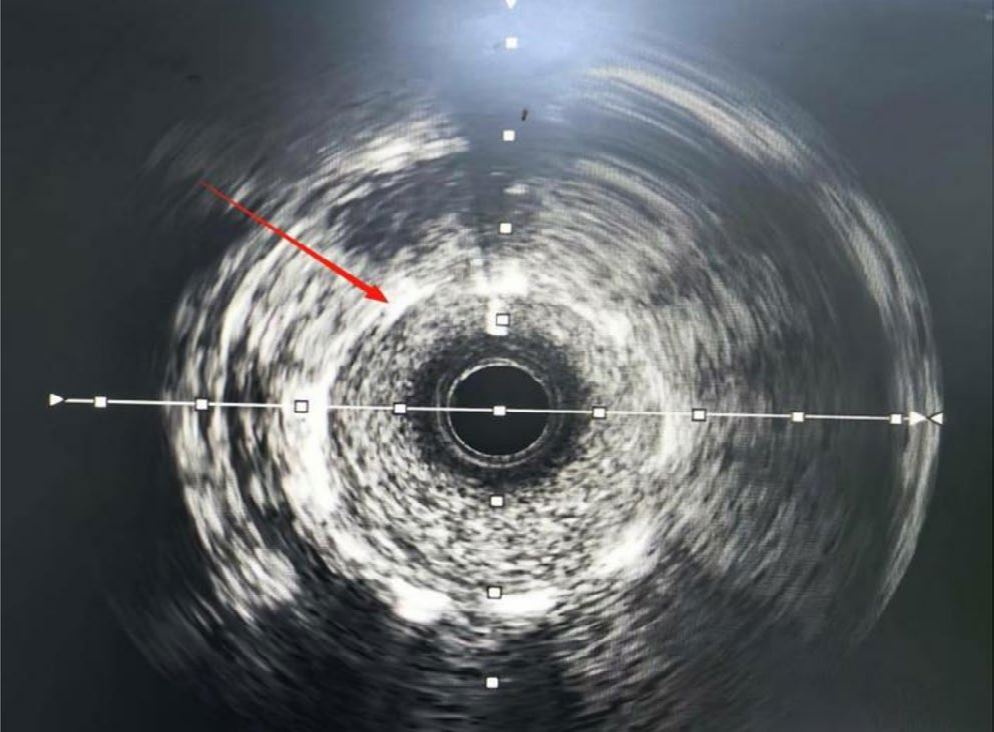
Coronary artery intravascular ultrasound image showing optimal stent expansion and good apposition to the vessel wall following intervention.

**FIGURE 5:**
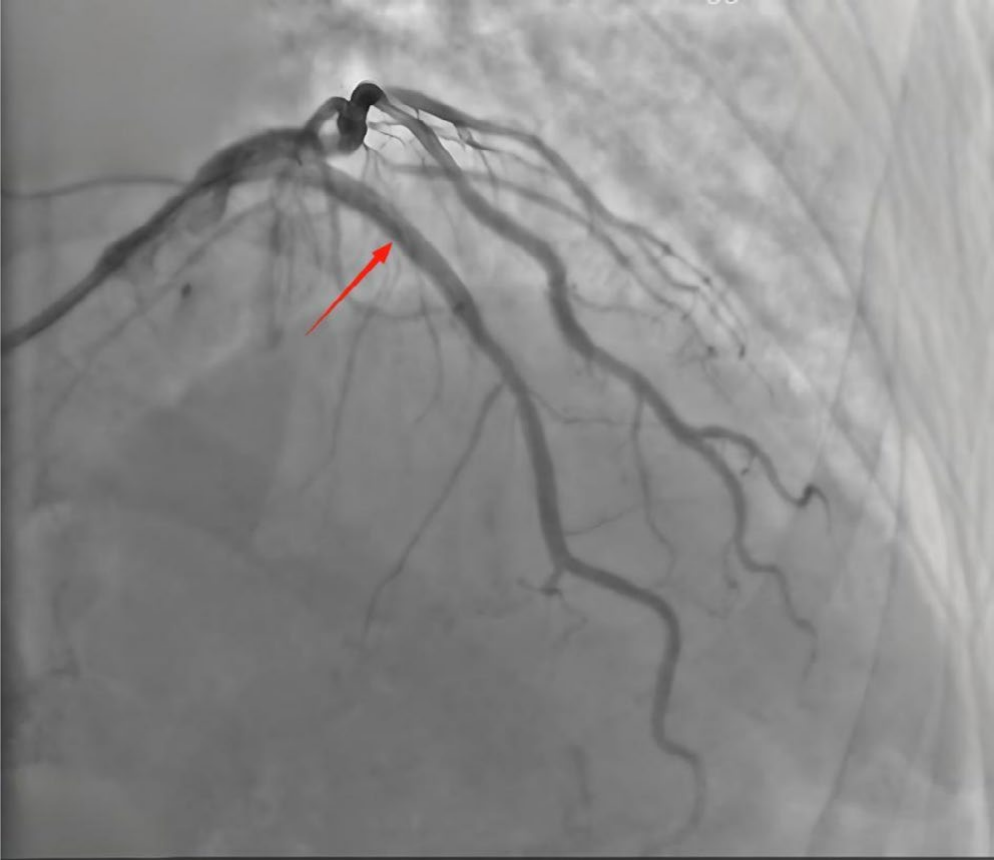
Coronary artery angiography after intravascular lithotripsy, demonstrating good stent expansion and proper positioning within the vessel.

After a failed angioplasty attempt with a 2.5 × 15‐mm noncompliant balloon (12–16 atm), IVL was performed using a 2.75 × 12‐mm catheter (SI‐SC001‐27512; Shenzhen Saihe Medical Technology Co. Ltd., Shenzhen, China). A balloon catheter with a diameter matching 0.8–1.0:1 of the reference vessel was selected. It was delivered to the calcified lesion site over a conventional workhorse guidewire and underwent seven treatment cycles. The balloon was inflated to 4 atm via a pressure pump to ensure close apposition with the vessel wall, and the lithotripsy generator was activated to deliver intermittent pulses. Each cycle consisted of 10 pulses delivered over 10 s. Each pulse generated a transient burst of high‐energy acoustic pulses, which traversed the coronary tissue and selectively disrupted the calcified lesion. This process selectively fractured both intimal and submedial calcifications. Following each therapeutic cycle, the IVL catheter was further expanded to 6 atm and maintained for 5–10 s to maximize vascular wall compliance and lumen patency. The modification effect on the calcified lesion was assessed by evaluating balloon symmetry during inflation. Subsequently, the balloon was depressurized and retracted to restore blood flow. If the desired therapeutic effect at a given lesion site was not achieved, multiple additional pulse cycles were delivered until satisfactory lumen gain was confirmed angiographically. For diffuse long lesions requiring staged treatment, the distal segment was treated first. When repositioning the catheter to treat the proximal adjacent segment, a 2‐mm overlap with the previously treated segment was ensured to prevent geographic miss. Following IVL, post‐procedural IVUS demonstrated successful calcified plaque modification (Figure 3) and optimal stent expansion (Figure 4). Subsequently, a 3.0 × 38‐mm stent (Resolute Integrity; Medtronic, Galway, Ireland; selected for its proven efficacy in complex lesions) was successfully deployed, achieving complete lesion coverage. Final angiography showed no residual stenosis (Figure 5).

#### 2.1.4 Outcome and Follow‐Up

The patient maintained clinical stability throughout the perioperative period and was discharged on the third postoperative day. At the 1‐year outpatient follow‐up, the patient reported no discomfort, with a follow‐up echocardiogram showing an ejection fraction (EF) of 61%.

### 2.2 Case 2

A 76‐year‐old woman presented on March 13, 2024, with recurrent chest pain for 1 month. Her medical history included hypertension, diabetes mellitus, and a history of PCI.

#### 2.2.1 Diagnostic Evaluation

Vital signs included temperature 36.8°C, pulse 80 bpm, RR 18 breaths/min, BP 137/75 mmHg. Cardiovascular examination revealed regular rhythm and no murmurs. Her lungs were clear bilaterally. Her abdomen was soft, nontender, and with no organomegaly. Neurological and musculoskeletal examinations revealed no edema or deficits.

#### 2.2.2 Cardiac Studies

**FIGURE 6:**
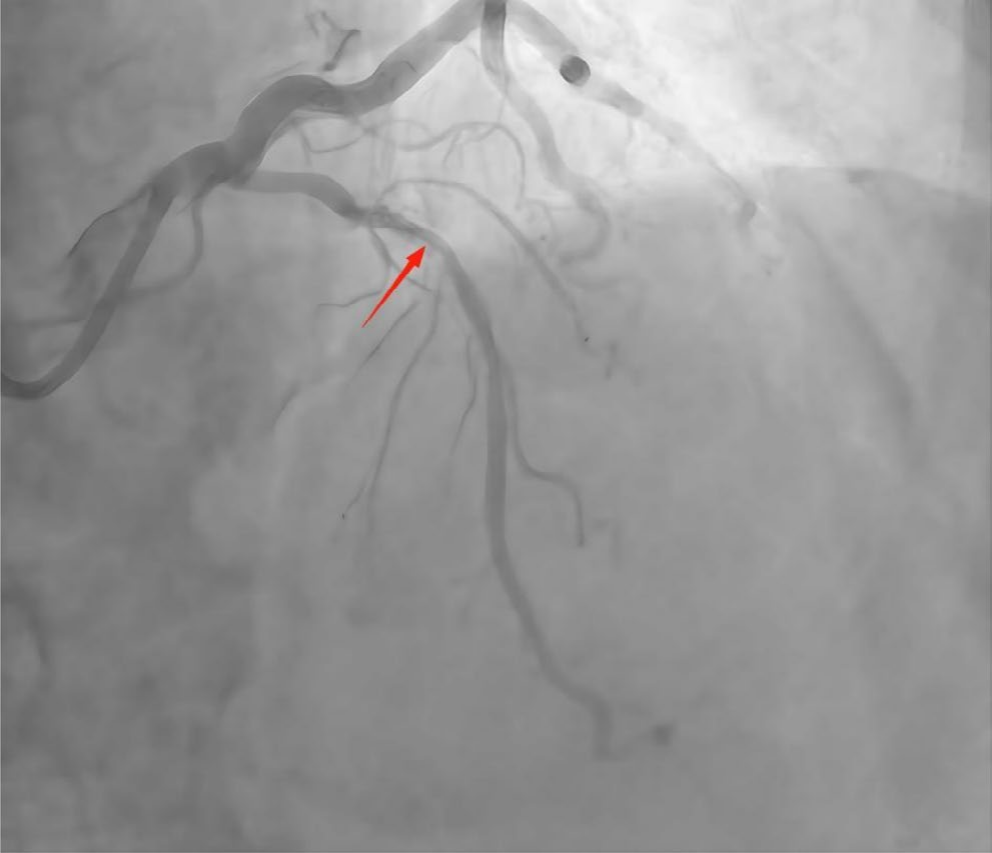
Preoperative coronary artery angiography demonstrating severe stenosis of the left anterior descending artery.

**FIGURE 7:**
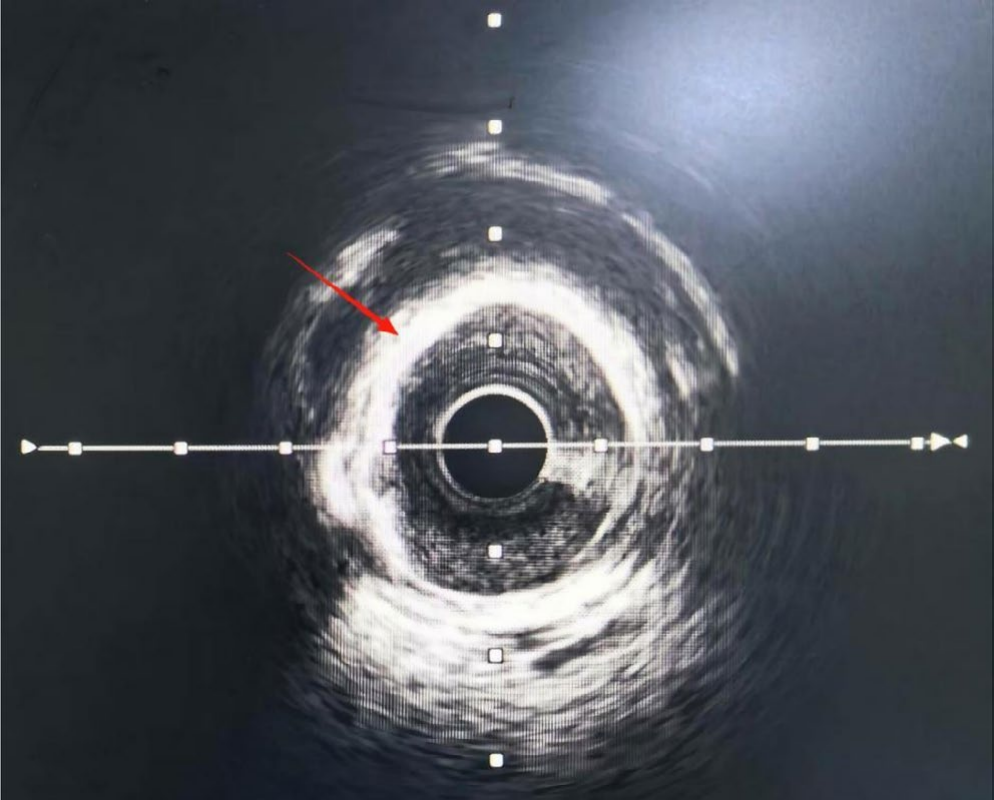
Intravascular ultrasound imaging of the coronary artery showing 270°–360° circumferential calcification.

ECG: Sinus rhythm and nonspecific ST‐T changes. TTE: Mild aortic/mitral regurgitation; left atrial enlargement; LVEF: 66%. Coronary angiography findings: The left main coronary artery (LM) showed no stenosis. LAD: Diffuse 70%–80% calcified stenosis in the proximal‐to‐mid segment (Figure 6). Diagonal branch: 85% stenosis. LCX: Tortuous but no significant stenosis. RCA: Proximal stent with 20% in‐stent restenosis and 40% stenosis at the distal stent edge. IVUS: Proximal‐to‐mid LAD, 270°–360° annular calcification; minimum lumen area 3.12 mm^2^ and stenosis rate 70% (Figure 7).

#### 2.2.3 Intervention

**FIGURE 8:**
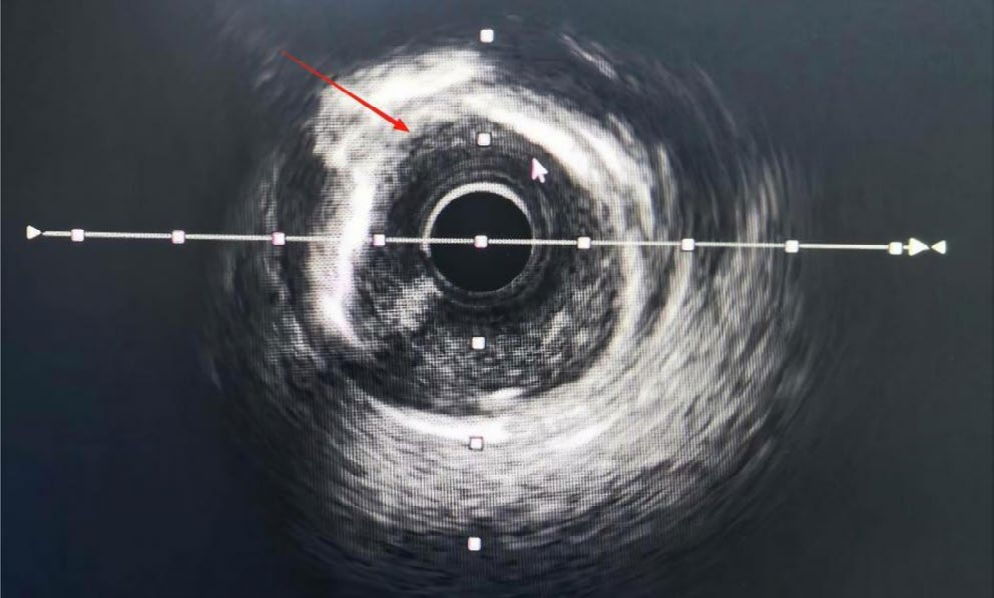
Intravascular ultrasound image showing fragmentation of calcified plaque following intravascular lithotripsy.

**FIGURE 9:**
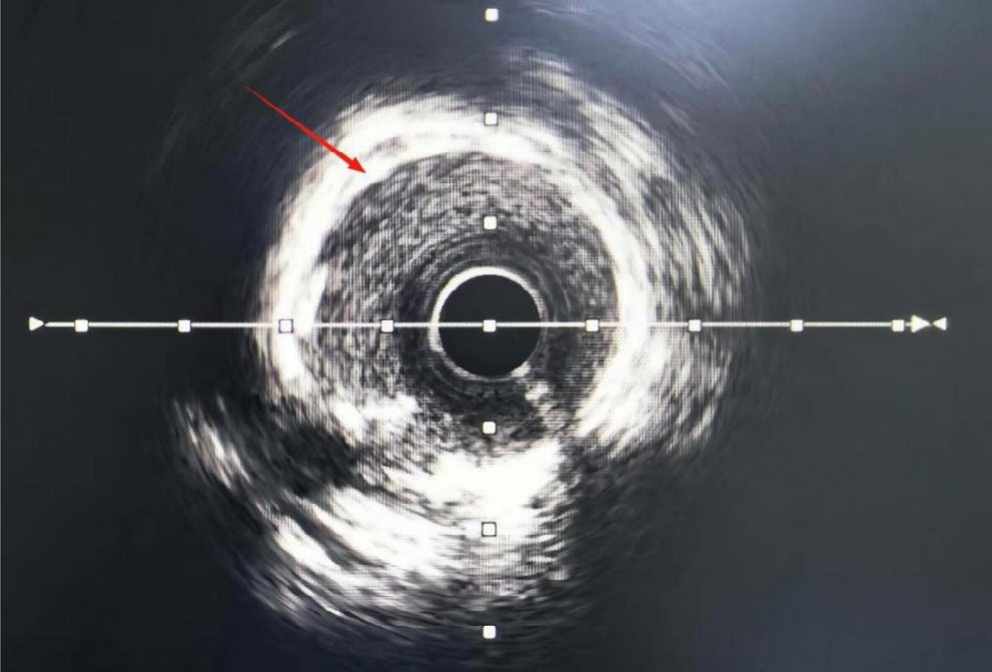
Intravascular ultrasound image demonstrating good stent expansion and vessel wall apposition following implantation.

**FIGURE 10:**
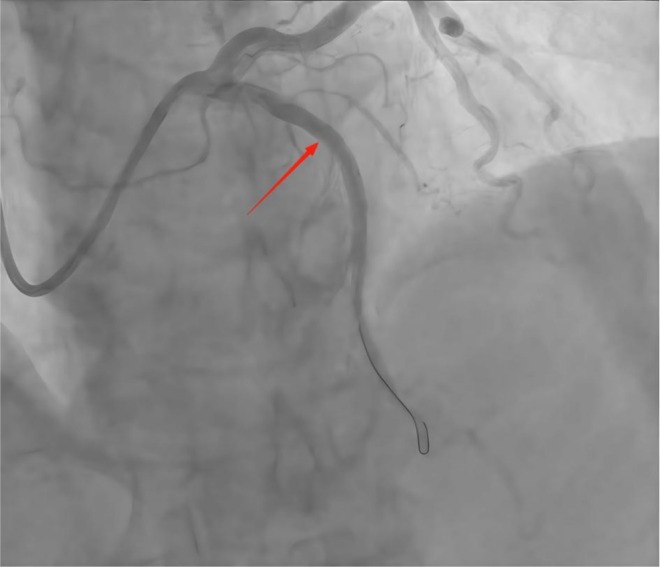
Coronary artery angiography demonstrating good stent expansion following intravascular lithotripsy.

Following lesion preparation with cutting balloon angioplasty for calcific resistance, IVL was performed using a 3.0 × 12‐mm balloon catheter (SI‐SC001‐27512; Shenzhen Sai he Medical Technology). A balloon catheter with a diameter matched 1:1 to the reference vessel was selected. Eight treatment cycles were performed, with each cycle delivering 10 pulses over a duration of 10 s. Post‐IVL IVUS confirmed calcified plaque fragmentation (Figure 8). Subsequently, two overlapping 3.0 × 28‐mm Promus Premier drug‐eluting stents (Boston Scientific, Marlborough, MA, USA) were deployed in the proximal‐to‐mid LAD artery with a 3‐mm overlap, followed by high‐pressure post‐dilation (3.0 × 12‐mm noncompliant balloon). IVUS demonstrated optimal stent expansion and apposition (Figure 9). Final angiography showed satisfactory stent deployment without dissection, with Thrombolysis in myocardial infarction (TIMI) grade 3 flow (Figure 10).

#### 2.2.4 Outcomes

The patient maintained clinical stability throughout the perioperative period and was discharged on the third postoperative day. At the 1‐year clinical follow‐up, the patient reported no discomfort, with a follow‐up echocardiography showing an EF of 63%.

### 2.3 Comparison of Procedural Characteristics Between the Two Patients

Preoperatively, both patients received dual antiplatelet therapy with a loading dose and were approached via the right radial artery. Anticoagulation during the procedure was achieved using unfractionated heparin injection (100 U/kg). Approximately 70 mL of contrast agent was used per patient. There was no requirement for temporary pacing or other device support during the procedures. Postoperatively, myocardial injury biomarkers (values measured within 24 h after the procedure) were elevated in both patients; however, no electrocardiographic evolution occurred. This finding does not exclude an association with sympathetic activation during PCI or myocardial ischemia caused by transient blood flow interruption during IVL. However, neither reported symptoms such as chest tightness, chest pain, or shortness of breath. No in‐hospital MACE, including death or target vessel revascularization, occurred. Both patients were discharged on the third postoperative day.

Table 1 quantitatively characterizes the baseline demographics, high‐risk calcified lesion morphology, and optimal procedural outcomes after IVL‐facilitated PCI.

**TABLE 1:** Baseline demographics, lesion characteristics, and procedural outcomes.

Parameter	Case 1	Case 2
**Demographics**		
Age/Sex	85‐year‐old woman	76‐year‐old woman
Clinical presentation	Chest tightness, dizziness	Recurrent angina
**Lesion characteristics**		
Target vessel/segment	Proximal/mid LAD	Proximal/mid LAD
Stenosis severity (%)	85	70–80
MLA (mm^2^)/plaque burden	2.66/71%	3.12/70%
Calcium arc	360°	270°–360°
**Key outcomes**		
Stent expansion (calculated as MSA [mm^2^]/mean reference lumen area [mm^2^]) (%)	5.93/5.97 = 99.33%	7.29/7.90 = 92.28%
Perioperative cTnI (μg/L)	1.244	0.372
Follow‐up LVEF	61%	63%
Final TIMI flow	Grade 3	Grade 3
Complications	None	None

Abbreviations: cTnI, cardiac troponin I; LAD, left anterior descending artery; LVEF, left ventricular ejection fraction; MLA, minimal lumen area; MSA, minimal stent area; TIMI, thrombolysis in myocardial infarction.

## 3 Treatment

For both patients, adequate antiplatelet therapy was administered preoperatively (enteric‐coated sustained‐release aspirin tablets, 300 mg; clopidogrel tablets, 300 mg). During hospitalization, standard secondary prevention of coronary heart disease was prescribed, which included enteric‐coated aspirin tablets (100 mg, once daily), clopidogrel tablets (75 mg, once daily), atorvastatin calcium tablets (20 mg, once nightly), metoprolol tablets (12.5 mg, twice daily), ezetimibe tablets (10 mg, once daily), and ACEI/ARB (angiotensin‐converting enzyme inhibitors/angiotensin II receptor blockers). The planned duration of dual antiplatelet therapy (DAPT) was 12 months for both patients. One year after discharge, both patients remained asymptomatic, reporting no chest tightness, chest pain, or shortness of breath, and were classified as New York Heart Association (NYHA) Class I.

## 4 Discussion

As shown in Figures 1, 2, 3, 4, 5, 6, 7, 8, 9, 10, IVL achieved consistent and favorable outcomes in both cases, highlighting its efficacy in the treatment of complex, highly calcified coronary lesions. The selection of IVL for these specific lesions was based on three key factors:

- Presence of 270°–360° circumferential calcification resistant to noncompliant/cutting balloons.
- Deep calcium deposits that were likely to prevent adequate stent expansion.
- Complications associated with rotational atherectomy (RA), including burr entrapment, coronary artery perforation, and no‐reflow; compared with RA, IVL offers a higher safety profile and superior cost‐effectiveness.

Coronary artery calcification is an important cause of adverse cardiovascular events [10]. For coronary artery calcification, contemporary plaque modification strategies include IVL, rotational/orbital atherectomy, and excimer laser coronary angioplasty (ELCA) [4]. IVL was prioritized over other plaque modification strategies because of its unique capacity to fracture both superficial and deep calcium layers without causing thermal injury or distal embolization—a critical advantage in lesions with extensive circumferential calcification (> 270°) [11, 12]. Effective calcium modification was confirmed by IVUS (Figures 3 and 8); optimal stent expansion was achieved (< 10% residual stenosis); no procedural complications occurred, including coronary perforation, no‐reflow, or stent malapposition. These results are consistent with multicenter registry evidence. The DISRUPT CAD IV trial reported a 92.2% rate of freedom from MACE at 1 year [13]. According to current expert consensus, IVL is recommended as one of the effective treatment modalities for specific lesion morphologies such as those with a large arc, deeply calcified nodules, or resistance to balloon angioplasty [14].

The present cases add to the growing evidence base by illustrating the clinical utility of IVL in elderly patients with severe calcification and complex anatomy, particularly when standard balloon techniques fail to achieve adequate lesion preparation. Nevertheless, this study has certain limitations. Although the short‐term prognosis was favorable, the single‐center, two‐case design, female sex and older ages of the patients, as well as the limited follow‐up period restrict generalizability. Because follow‐up was limited to 1 year, assessment of long‐term stent patency and restenosis rates was not possible. Although current evidence has demonstrated a favorable efficacy and safety profile for IVL in the short term, longer‐term monitoring and additional case studies are warranted to better understand the prognosis and to inform management strategies for similar patients. Further studies should include larger, multicenter, prospective randomized trials to directly compare IVL with alternative plaque modification techniques, particularly RA, in terms of long‐term efficacy, stent durability in calcified segments, and cost‐effectiveness.

## Author Contributions

**Liang‐cai Guo:** conceptualization, data curation, formal analysis, investigation, methodology, writing – review and editing. **Chi‐jun Hou:** data curation, methodology, validation, writing – original draft. **Yan‐jin Zeng:** data curation, methodology, validation, writing – original draft. **Wen Li:** data curation, methodology, validation.

## Funding

The authors have nothing to report.

## Ethics Statement

This case series was conducted in accordance with local institutional policies; institutional review board approval was not required for single‐center case reports per local regulations.

## Consent

Written informed consent was obtained from each patient for publication of clinical details and images.

## Conflicts of Interest

The authors declare no conflicts of interest.

## Data Availability

The data that support the findings of this case study were available within the manuscript.
